# Neonatal Tetanus Immunity in Nigeria: The Effect of HIV Infection on Serum Levels and Transplacental Transfer of Antibodies

**DOI:** 10.1155/2016/7439605

**Published:** 2016-01-20

**Authors:** Muhammad Faruk Bashir, Hassan Abdullahi Elechi, Mohammed Garba Ashir, Adamu Ibrahim Rabasa, David Nadeba Bukbuk, Ahmadu Baba Usman, Modu Gofama Mustapha, Mohammad Arab Alhaji

**Affiliations:** ^1^Department of Paediatrics, Abubakar Tafawa Balewa University Teaching Hospital, Bauchi 740222, Nigeria; ^2^Department of Paediatrics, University of Maiduguri, Maiduguri 600243, Nigeria; ^3^Department of Microbiology, University of Maiduguri, Maiduguri 600243, Nigeria; ^4^Department of Paediatrics, Federal Medical Centre Yola, Yola 640101, Nigeria

## Abstract

*Background*. Tetanus toxoid immunisation of pregnant mother has remained the most effective strategy in eliminating neonatal tetanus. Impaired production and/or transplacental transfer of antibodies may affect the effectiveness of this strategy. We studied the effect of maternal HIV infection on serum levels and transplacental transfer of anti-tetanus antibodies.* Methods*. A total of 162 mother-baby paired serum samples were taken and analysed for anti-tetanus antibody levels using ELISA. Maternal HIV status was also determined by double ELISA technique. Maternal TT vaccination status was also documented.* Results*. Thirty-eight (23.5%) mothers and 41 (25.3%) babies were seronegative, out of whom 8 mothers were HIV positive and 9 babies were HIV exposed. HIV infected mothers and HIV exposed infants were, respectively, 16.27 times (OR = 16.27, 95% CI = 3.28 to 80.61) and 33.75 times (OR = 33.75, 95% CI = 4.12 to 276.40) more likely to be seronegative for anti-tetanus antibody. Similarly, HIV positive mother-newborn pairs were 7.46 times more likely to have a poor transplacental transfer of tetanus antibodies (OR = 7.46, 95% CI = 1.96 to 28.41).* Conclusions*. Maternal HIV infection is associated with impaired maternofoetal transfer of anti-tetanus antibodies and seronegativity among mothers and their newborns. Hence, this may hinder efforts to eliminate neonatal tetanus.

## 1. Introduction

In developing countries, Human Immunodeficiency Virus (HIV) infection and neonatal tetanus (NT) are among the leading causes of childhood morbidity and mortality. Understanding the interaction between HIV and NT is important since it may have a major implication for NT elimination program. Newborns are protected from NT by maternal anti-tetanus antibody (MTA) of the IgG class which is actively transferred across the placental barrier from third trimester of gestation following tetanus toxoid (TT) vaccination in pregnant women. Strengthening of immunisation for pregnant women with TT vaccine is reported to be the single most effective strategy independent of other interventions in eliminating neonatal tetanus [1]. Its efficacy and effectiveness has been convincingly demonstrated in many field trials and in hospital-based studies [2–6].

However, there are conflicting reports of the influence of maternal HIV infection on anti-tetanus antibody production by the mothers as well as its transfer through the placenta to their unborn babies. Whereas lower anti-tetanus antibody levels in HIV infected women were reported from Senegal [7], Brazil [8], and The Gambia [9], no such relationship was found in Malawi [10]. Similarly, Cumberland et al. [11] in Kenya reported a decrease in cord-maternal ratio (CMR) with 50% reduction in cord blood tetanus antibody concentration in babies born by HIV infected mothers. Similar conclusions were arrived at by Bonetti et al. [8] in Brazil. To the best of our knowledge, no such study was carried out in Nigeria which happens to be one of the 46 remaining countries yet to eliminate neonatal tetanus as at 2008 and among the 24 countries responsible for 90% of the global burden of the disease [12]. Moreover, Chama et al. in 2010 reported a high prevalence of HIV infection of 12.7% among women attending antenatal clinic of the University of Maiduguri Teaching Hospital (UMTH), Maiduguri, northeastern Nigeria, where this study was carried out. We thus aim to assess the effect of maternal HIV infection on the serum levels of anti-tetanus antibodies in mother-infant pairs and hence transplacental transfer of antibody.

## 2. Materials and Method

### 2.1. Study Area

The study was carried out at the Labour Ward of the UMTH, Nigeria. The UMTH is a tertiary-based care centre located in northeastern Nigeria and a centre of excellence for infectious diseases and immunology. It also serves as a referral site for the six northeastern states of Nigeria and the neighboring countries of Chad, Cameroon, and Niger.

### 2.2. Design

The study was a hospital-based descriptive and cross-sectional study of mother-newborn pairs recruited from the Labour Ward of the UMTH.

### 2.3. Ethical Consideration

The study protocol was reviewed and approved by the Medical Research and Ethics Committee of UMTH. Informed consent of the delivering other was also obtained. Participants had unlimited liberty to deny consent without any consequences and confidentiality was maintained.

### 2.4. Sample Size

The minimum sample size was determined using Cochran's sample size formula for categorical data [13] at alpha level of 0.05 and power of 95%. Proportion of newborns with protective level of tetanus IgG antibody (seropositives), value of *p*, was taken from a previous study in Jos by Adabara et al. [14] to be 88%. Therefore, the sample size for the study was one hundred and sixty-two mother-newborn pairs.

### 2.5. Data Collection

Mother-newborn pairs were enrolled in this study using the systematic random sampling method, in which the first of every three mother-newborn pairs was picked at the Labour Ward from the first day of data collection after fulfilling the eligibility criteria. Exclusion criteria were failure to give consent (0.05%), stillbirth and history of antitetanus serum (ATS), or blood transfusion during pregnancy. Mothers with diabetes mellitus, hypertension, eclampsia, or preeclampsia were also excluded. Upon enrolment, a questionnaire detailing sociodemographic variables was completed for each mother-newborn pair. This included biodata, pregnancy history and antenatal history, and gestational age as well as indicators of socioeconomic status like occupation and educational levels of both parents. History of TT vaccination during current pregnancy, verifiable from the patients' antenatal clinic records, where possible, was obtained, including how many doses and their interval as well as TT received before the index pregnancy (both during and outside pregnancy). Gestational age (GA) at delivery was determined by date using the last menstrual period or by first-trimester ultrasound scan, where available. Dubowitz Score was used to access the GA of the newborn infants at birth [15], and their birth weight (BW) was measured using a digital weighing scale that has a sensitivity of 0.01 kg. Newborn infants with GA less than 37 completed weeks were classified as preterm, those from 37 completed to less than 42 completed weeks were term, and those with 42 completed weeks or more were postterm [15]. Newborns weighing >3.99 kilograms were classified as macrosomia, those weighing 2.5–3.99 kilograms were classified as normal, and those <2.5 kilograms were termed low BW [15]. Socioeconomic status was assigned using Oyedeji's model [16].

### 2.6. Sample Collection and Processing

Maternal venous blood (2 mL) was collected from a peripheral vein following aseptic procedure. Two millilitres of cord blood from the placental end of the cord, after early clamping of the cord, was also collected and stored in a properly labelled sterile plain bottle. Sera were separated after centrifuging these blood samples at 5000 rpm for five minutes. Aliquots of serum samples were stored at −20°C until analysis. All mother-newborn pairs sera were assayed for tetanus IgG antibodies by Enzyme Linked Immunosorbent Assay (ELISA) technique [17]. The optical density at 450 nm (OD_450_) was measured in an ELISA microplate reader. Standard curves were drawn for each plate and optical densities of the test serum dilutions falling within the linear part of the curve were extrapolated. The results were expressed as IU per millilitre. World Health Organization (WHO) guidelines proposed a cut-off of 0.1–0.2 IU/mL for a protective antibody concentration [18]. For this study, antibody level of 0.1 IU/mL was used as the cut-off, below which individuals were classified as being seronegative.

Maternal HIV status was defined by presence of serum HIV antibodies determined by two different ELISA tests, as recommended by WHO [19]. Any two of Determine, Stat-pack, or Unigold were used.

### 2.7. Data Analysis

Data obtained were entered into a computer that generated a computerized data base. Analysis was done using SPSS version 16.0 (SPSS, Chicago, Illinois, USA). Tables were used for data presentation as appropriate. Geometric means ± standard deviations of tetanus antibody titres were determined. Frequencies of tetanus antibody serostatus (seropositives and seronegatives) were compared using Fisher exact test. A *p* value of <0.05 was considered significant.

## 3. Results

A total of 162 mother-newborn pairs were enrolled into this study. The mean ± standard deviation (SD) age of the mothers was 27.2 ± 6.0 years. Most of them were multipara (121 [74.7%]) and belonged to the low socioeconomic class (132 [81.5%]). One hundred and thirty-eight (85.2%) mothers had at least 2 tetanus toxoid (TT) vaccinations during the current pregnancy. Ten (6.2%) mothers were HIV infected and were on antiretroviral treatment (Table 1). Nine (90%) of the HIV positive mothers had at least 2 doses of TT vaccination; the remaining woman had only a single dose. There was no significant variation in doses of TT vaccination between the HIV positive and HIV negative mothers (*p* = 1.000).

Among the 162 babies enrolled in the study, 84 (51.9%) were males and 78 (48.1%) were females with a male to female ratio (M : F) of 1.08 : 1. Most of them, 149 (92%), were term and the remaining 13 (8%) were preterm. None of the babies was postterm. Low birth weight was observed in 9 (5.6%) babies while the remaining 153 (94.4%) were of normal birth weight.

**Figure 1:**
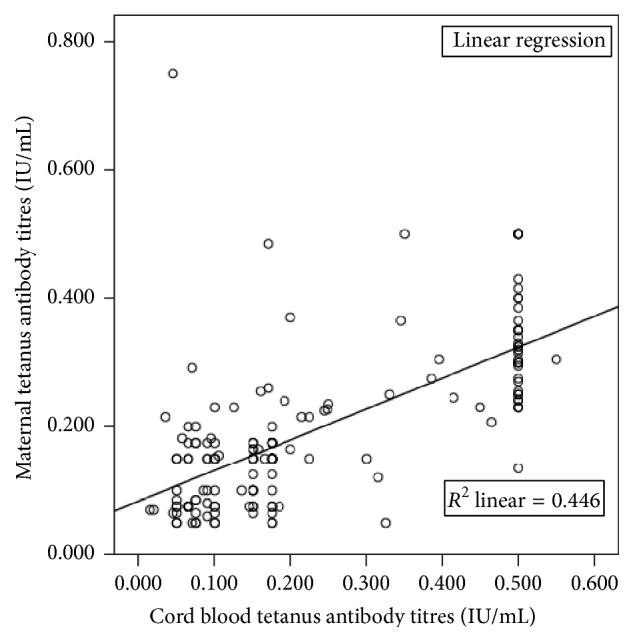
Pearson correlation between maternal and cord blood anti-tetanus antibody titres.

One hundred and twenty-four mothers (76.5%) and 121 (74.4%) babies were seropositive for tetanus antibodies (titre of ≥0.1 IU/mL). The remaining were seronegative (titre <0.1 IU/mL), out of whom 8 of the mothers were HIV positive and 9 of the babies were delivered by HIV positive mothers. The mean (SD) tetanus antibody titres of the studied mothers and their babies were 0.160 (0.122) and 0.230 (0.170) IU/mL, respectively. There was a significant strong positive correlation between maternal and cord blood levels of tetanus antibodies (*r* = +0.668, *r* ^2^ = 0.445, *p* < 0.001) with a shared variance of 44.6% (Figure 1).

**Figure 2:**
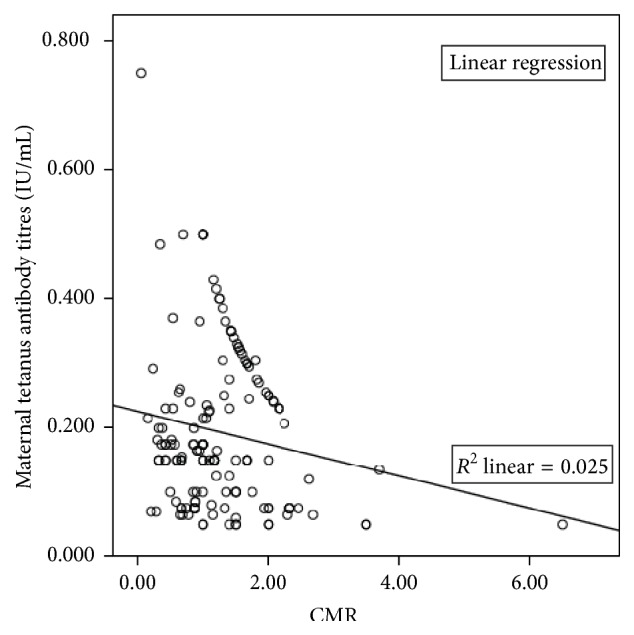
Pearson correlation between maternal anti-tetanus antibody titres and cord-maternal ratios (CMR).

One hundred and six pairs (65.4%) had cord-maternal ratios (CMR) of ≥1 with a mean (SD) of 1.499 (0.729), while the remaining 56 (34.6%) had ratios of <1 with a mean (SD) of 0.553 (0.232). The overall mean (SD) of the cord-maternal ratios was 1.062 (0.767). There was a significant but weak negative correlation between maternal tetanus antibody levels and cord-maternal ratios (*r* = −0.158, *p* = 0.045) with a shared variance of only 2% (Figure 2).

On the effects of the various maternal and neonatal factors on the anti-tetanus antibody serostatus and CMR, only maternal TT vaccination and HIV status showed statistically significant association with maternal anti-tetanus antibody serostatus (*p* ≤ 0.001) for both. Similarly, HIV exposure and gestational age at birth were the only factors with statistically significant association with cord anti-tetanus antibody serostatus (*p* ≤ 0.001 and 0.004), respectively. In both cases, these factors were still found to have a significant predictive value even after controlling for all the other variables using logistic regression analysis (Tables 2 and 3) for maternal serostatus and cord serostatus, respectively. Maternal HIV infection and neonatal gestational age at birth were the only factors with statistically significant association with CMR (*p* = 0.001 and 0.033) for maternal HIV and gestational age, respectively.

HIV infected mothers were 16.27 times more likely to have seronegative levels of tetanus antibody than HIV negative mothers. Similarly, HIV exposed babies were 33.75 times more likely to have seronegative cord blood than those delivered by HIV negative mothers. Again, HIV positive mother-newborn pairs were 4.91 times more likely to have a poorly efficient transplacental transfer of tetanus antibodies with CMR of <1.0 compared to HIV negative pairs (Table 4).

## 4. Discussion

The observed HIV prevalence of 6.2% among the mothers in this study is quite above the national average of 3.7% [20] This higher prevalence may be due to several factors. The UMTH antenatal clinic serves as a referral centre for HIV positive pregnant women from lower cadre health facilities for the purpose of enrolment into the PMTCT program. This will undoubtedly lead to concentration of HIV positive pregnant women in this hospital and hence the observed higher prevalence. Secondly, the child bearing age also corresponds to the age of maximal sexual activity and has been found to have the highest incidence of HIV infection [21]. This prevalence is, however, about half of 12.7% reported by Chama et al. [22] in 2010 among women attending antenatal clinic from the same hospital where this study was carried out. The lower prevalence observed in this study, compared to the one by Chama et al. [22] 4 years earlier, could be a reflection of the general decline in HIV prevalence being experienced in the country [23]. Secondly, while the study by Chama et al. considered all women attending ANC in UMTH over a 2-year period, this study enrolled 1 of every 3 women that delivered in UMTH over a period of 6 months until the required sample size was attained. Therefore, the difference in sampling technique might be a contributing factor.

The observed overall protective levels of tetanus antibodies (mean ≥0.1 IU/mL) for both mothers and infants are similar to the report of Cumberland et al. [11] in Kenya in 2007. They found that the geometric mean titres of both the mothers and their babies were within the protective range. Similarly, Hood et al. [24] found the geometric mean concentration of tetanus antibodies in Nigerian mothers and their infants to be within the protective range. The high proportion of tetanus antibody seropositivity among both the mothers and their babies in this study is similar to the findings in earlier studies from Kenya [11], Jos [14], and Ibadan [24]. Adabara et al. [14] at the Jos University Teaching Hospital, Nigeria, in 2010 also reported a highly significant positive correlation between the maternal tetanus antibody levels and those of the babies similar to our finding. These findings, however, are probably overestimates of the mean tetanus antibody titre and proportion of seropositivity in the general population, because women who deliver in hospital are more likely to have received TT vaccination than women who deliver at home [25], hence further underscoring the import of TT vaccination in attainment of anti-tetanus antibody seropositivity.

The mean cord-maternal ratio found in this study was higher than most previous studies reported from Kenya [11]; Ibadan, Nigeria [24]; Libreville, Gabon [26], as well as Thailand [27] and India [28]. While it was found to be >1.0 in this study signifying efficient placental transfer, the others reported <1.0, pointing to the earlier held view of poor placental transfer of tetanus antibodies in African mothers. However, besides the small sample size in the Ibadan study, most of the studies used* in vitro* technique to determine tetanus antibody levels which measures both IgG and IgM antibodies resulting in misleadingly high maternal titres and therefore low cord-maternal ratios, compared with standard ELISA (*in-vivo*) used in this study which measures only IgG.

The effect of maternal HIV infection on maternal serum and cord tetanus antibody levels as well as transplacental transfer of this antibody in this study is consistent with the findings of lower tetanus antibody levels in HIV infected women in Senegal [7], Brazil [8], and The Gambia [9]. It is also in agreement with data from Kenya, in the largest study of the association between maternal HIV infection and tetanus antibody levels by Cumberland et al. [11], where they found lower levels of tetanus antibody among HIV infected women, reduced transplacental transfer of tetanus antibody, and ~50% lower antibody levels in cord serum. In contrast, De Moraes-Pinto et al. from Malawi found that maternal HIV infection had no effect on maternal serum and cord anti-tetanus IgG levels as well as transplacental transfer of this antibody [10]. The reason for this contrasting finding is not clear but may be due to the fact that the authors did not adjust for TT vaccination status which has been demonstrated in this study and others [2–6] to be an important determinant of serum tetanus antibody status.

## 5. Conclusion

Maternal HIV infection was associated with significantly impaired maternofoetal transfer of tetanus antibody and seronegativity (unprotective) levels in both mother-newborn pairs at birth despite being on antiretroviral therapy for HIV. Hence, this may hinder efforts to eliminate neonatal tetanus. There is need to review and restrategize neonatal tetanus elimination program among this vulnerable population. Such strategies may include passive immunisation in addition to other elements of prevention among high risk groups.

## Figures and Tables

**Table 1:** Sociodemographic and obstetric characteristics of the mothers studied.

Characteristic	Frequency (*N* = 162)	Percent
Age (years)		
14–19	14	8.6
20–24	38	23.5
25–29	54	33.3
≥30	56	34.6
Mean (SD)	27.2 (6.0)	
Parity		
Primipara	41	25.3
Multipara	121	74.7
Socioeconomic status		
High	14	8.6
Middle	16	9.9
Low	132	81.5
TT vaccination status		
None	12	7.4
1	12	7.4
≥2	138	85.2
HIV status		
Positive	10	6.2
Negative	152	93.8

SD = standard deviation; TT = tetanus toxoid.

**Table 2:** Logistic regression analysis results exploring the predictive significance of maternal factors on maternal tetanus antibody serostatus.

Independent variables	Wald test	df	*p* value
Maternal age	0.876	3	0.831
SES	1.095	2	0.579
Parity	0.067	1	0.795
TT vaccination status	10.161	2	0.006^x^
HIV status	9.581	1	0.002^x^
Placental malaria	0.770	1	0.380
Constant	4.110	1	0.043

SES = socioeconomic status; df = degree of freedom; ^x^significant relationship.

**Table 3:** Logistic regression analysis results exploring the predictive significance of some maternal and infant factors on cord-blood tetanus antibody serostatus.

Independent variables	Wald test	df	*p* value
Maternal age	2.050	3	0.562
SES	3.052	2	0.217
Parity	0.386	1	0.534
TT vaccination status	0.141	2	0.932
HIV status	10.950	1	0.001
Placental malaria	1.312	1	0.252
Birth weight	0.063	1	0.802
Gestational age	7.859	1	0.005
Gender	0.545	1	0.460
Constant	0.165	1	0.685

SES = socioeconomic status; df = degree of freedom; *p* significant at <0.05.

**Table 4:** The effect of HIV infection on maternal and newborn anti-tetanus antibody status and cord-maternal ratio (CMR).

HIV status	Anti-tetanus antibody status		OR	CI	*p* value
	Seronegative (<0.1)	Seropositive (≥0.1)			
Mothers					
HIV positive	8	2	16.27	3.28–80.61	<0.001^*∗*^
HIV negative	30	122			
Total	**38**	**124**			
Newborns					
HIV exposed	9	1	33.75	4.12–276.4	<0.001^*∗*^
Not exposed	32	120			
Total	**41**	**121**			

	Cord-maternal ratio				
	Inefficient transfer (<1)	Efficient transfer (≥1)			

Mothers					
HIV positive	7	3	4.91	1.22–19.79	0.033^*∗*^
HIV negative	49	103			
Total	**56**	**106**			

HIV, human immunodeficiency virus; OR, odds ratio; CI, confidence interval; ^*∗*^Fisher's exact test.

## Limitations

The study is hospital based and the studied population were skewed towards certain characteristics such as low socioeconomic class, multiparity, and term babies making generalization of the result difficult.

## References

[1] Gupta S. D., Keyl P. M. (1998). Effectiveness of prenatal tetanus toxoid immunization against neonatal tetanus in a rural area in India. *Pediatric Infectious Disease Journal*.

[2] Newell K. W., Duenas L. A., LeBlanc D. R., Garces Osorio N. (1966). The use of tetanus toxoid for the prevention of neonatal tetanus in developing countries for the prevention of tetanus neonatorum. *Bulletin of the World Health Organization*.

[3] Black R. E., Huber D. H., Curlin G. T. (1980). Reduction of neonatal tetanus by mass immunization of non-pregnant women: duration of protection provided by one or two doses of aluminium-adsorbed tetanus toxoid. *Bulletin of the World Health Organization*.

[4] Berggren G. G., Berggren W., Verly A. (1983). Traditional midwives, tetanus immunization, and infant mortality in rural Haiti. *Tropical Doctor*.

[5] Cliff J. (1985). Neonatal tetanus in Maputo, Mozambique Part II. Preventative measures. *Central African Journal of Medicine*.

[6] Vandelaer J., Birmingham M., Gasse F., Kurian M., Shaw C., Garnier S. (2003). Tetanus in developing countries: an update on the maternal and neonatal tetanus elimination initiative. *Vaccine*.

[7] Dieye T. N., Sow P. S., Simonart T. (2002). Immunologic and virologic response after tetanus toxoid booster among HIV-1 and HIV-2-infected Senegalese individuals. *Vaccine*.

[8] Bonetti T. C. S., Succi R. C. M., Weckx L. Y., Tavares-Lopes L., De Moraes-Pinto M. I. (2004). Tetanus and diphtheria antibodies and response to a booster dose in Brazilian HIV-1-infected women. *Vaccine*.

[9] Okoko B. J., Wesuperuma L. H., Ota M. O. (2001). Influence of placental malaria infection and maternal hypergammaglobulinaemia on materno-fetal transfer of measles and tetanus antibodies in a rural west African population. *Journal of Health, Population, and Nutrition*.

[10] De Moraes-Pinto M. I., Verhoeff F., Chimsuku L. (1998). Placental antibody transfer: influence of maternal HIV infection and placental malaria. *Archives of Disease in Childhood: Fetal and Neonatal Edition*.

[11] Cumberland P., Shulman C. E., Maple P. A. C. (2007). Maternal HIV infection and placental malaria reduce transplacental antibody transfer and tetanus antibody levels in newborns in Kenya. *Journal of Infectious Diseases*.

[12] WHO Immunisation, surveillance, assessment, and monitoring.

[13] Cochran W. G. (1977). *Sampling Techniques*.

[14] Adabara N. U., Kandakai-Olukemi Y. T., Enenebeaku M. N., Daru P. H. (2010). Assessment of materno-foetal transfer of antitetanus immunoglobulin G in Jos University Teaching Hospital, Jos. *Shiraz E Medical Journal*.

[15] Stoll B. J., Adams-Chapman I., Kliegman R. M., Behrman R. E., Jenson H. B., Stanton B. F. (2007). The high-risk infant. *Nelson Textbook of Pediatrics*.

[16] Oyedeji G. A. (1985). Socioeconomic and cultural background of hospitalized children in Ilesha. *Nigerian Journal of Paediatrics*.

[17] (2009). *Product Information Instruction Manual. Enzyme Immunoassay for the Detection and the Quantitative Determination of Human IGG Antibodies against Tetanus Toxoid in Serum and Plasma*.

[18] WHO (2006). Tetanus vaccine: WHO position paper. *The Weekly Epidemiological Record*.

[19] WHO (1992). Global program on AIDS—recommendations for the selection and use of HIV antibody tests. *Weekly Epidemiological Record*.

[20] Federal Republic of Nigeria (2012). *Global AIDS Response Country Progress Report, Nigeria*.

[21] Omisakin C. T., Esan A. J., Fasakin K. A. (2014). Syphilis and Human Immunodeficiency virus co-infection among pregnant women in Nigeria: prevalence and trend. *International STD Research & Reviews*.

[22] Chama C. M., Bello M., Ajayi B. A., Zarma S., Gashau W. (2010). The use of highly active antiretroviral therapy for the prevention of mother-to-child transmission of the human immunodeficiency virus in Nigeria. *Journal of Obstetrics and Gynaecology*.

[23] NACP (2010). National HIV prevalence sentinels survey among pregnant women in Nigeria.

[24] Hood N., Chan M. C. K., Maxwell S. M., Familusi J. B., Hart C. A. (1994). Placental transfer of tetanus toxoid antibodies in Nigerian mothers. *Annals of Tropical Paediatrics*.

[25] Cutts F. T., Rodriguez L. C., Colombo S., Bennett S. (1989). Evaluation of factors influencing vaccine uptake in Mozambique. *International Journal of Epidemiology*.

[26] Michaux J. L., Heremans J. F., Hitzig W. H. (1966). Immunoglobulin levels in cord-blood serum of negroes and Caucasians. *Tropical and Geographical Medicine*.

[27] Sangpetchsong V., Vichaikummart S., Vichitnant A., Podhipak A. (1984). Transfer rate of transplacental immunity to tetanus from non-immunized and immunized mothers. *Southeast Asian Journal of Tropical Medicine and Public Health*.

[28] Maselle S. Y. (1989). Maternal and foetal tetanus toxoid antibody levels following immunization in pregnancy. *Journal of Obstetrics and Gynaecology of Eastern and Central Africa*.

